# Endothelial Targeting of Cowpea Mosaic Virus (CPMV) via Surface Vimentin

**DOI:** 10.1371/journal.ppat.1000417

**Published:** 2009-05-01

**Authors:** Kristopher J. Koudelka, Giuseppe Destito, Emily M. Plummer, Sunia A. Trauger, Gary Siuzdak, Marianne Manchester

**Affiliations:** 1 Department of Cell Biology, The Scripps Research Institute, La Jolla, California, United States of America; 2 Center for Integrative Molecular Biosciences, The Scripps Research Institute, La Jolla, California, United States of America; 3 Dipartimento di Medicina Sperimentale e Clinica, Università degli Studi Magna Graecia di Catanzaro, Viale Europa, Campus Universitario di Germaneto, Catanzaro, Italy; 4 Department of Molecular Biology, The Scripps Research Institute, La Jolla, California, United States of America; 5 Center for Mass Spectrometry, The Scripps Research Institute, La Jolla, California, United States of America; University of California San Francisco, United States of America

## Abstract

Cowpea mosaic virus (CPMV) is a plant comovirus in the picornavirus superfamily, and is used for a wide variety of biomedical and material science applications. Although its replication is restricted to plants, CPMV binds to and enters mammalian cells, including endothelial cells and particularly tumor neovascular endothelium *in vivo*. This natural capacity has lead to the use of CPMV as a sensor for intravital imaging of vascular development. Binding of CPMV to endothelial cells occurs via interaction with a 54 kD cell-surface protein, but this protein has not previously been identified. Here we identify the CPMV binding protein as a cell-surface form of the intermediate filament vimentin. The CPMV-vimentin interaction was established using proteomic screens and confirmed by direct interaction of CPMV with purified vimentin, as well as inhibition in a vimentin-knockout cell line. Vimentin and CPMV were also co-localized in vascular endothelium of mouse and rat *in vivo*. Together these studies indicate that surface vimentin mediates binding and may lead to internalization of CPMV *in vivo*, establishing surface vimentin as an important vascular endothelial ligand for nanoparticle targeting to tumors. These results also establish vimentin as a ligand for picornaviruses in both the plant and animal kingdoms of life. Since bacterial pathogens and several other classes of viruses also bind to surface vimentin, these studies suggest a common role for surface vimentin in pathogen transmission.

## Author Summary

Cowpea mosaic virus (CPMV), a plant virus that does not replicate in animals, is extensively used in material science and nanobiotechnology. CPMV has been found to specifically interact with mammalian cells after oral or intravenous administration, as well as in intravital vascular imaging studies that used a fluorescently modified form of CPMV. Binding of CPMV to mammalian cells was shown to be via a cell-surface binding protein (CPMV-BP). Herein we identify this cell surface CPMV-BP through biochemical analysis, live cell experiments, and animal models. We found this surface exposed protein to be vimentin. Vimentin is principally a cytoskeletal protein that functions in the interior of cells to modulate architecture and dynamics. Our results now indicate surface vimentin can be used as a vascular endothelial marker and targeting option on the exterior surface of these cells. This work also unifies the relationship between CPMV and closely related mammalian viruses such as poliovirus, Theiler's murine encephalomyelitis virus (TMEV), and coxsackie virus through the collective use of vimentin during their infectious cycle. Several other bacterial and viral pathogens use surface vimentin as an attachment receptor as well, and this research may lead to the development of broad-spectrum strategies to inhibit infection.

## Introduction

Cowpea mosaic virus (CPMV) is a member of the *comoviridae* family of plant viruses. The 31 nm-diameter capsid has a pseudo T = 3 symmetry composed of 3 beta-barrel domains formed from 2 capsid proteins, and is structurally related to animal picornaviruses that include such viruses as poliovirus, coxsackievirus, and Theiler's murine encephalomyelitis virus (TMEV) [1]. Within the picornavirus-like superfamily these viruses also share a similar genetic organization and along with CPMV are thought to derive from a common ancestor [2],[3]. The mechanisms of evolution of the picorna-like viruses within the kingdoms of life, and possible cross-kingdom transmission during evolution, are unknown.

In addition to its role as a plant pathogen, CPMV has received recent attention as a nanoscale scaffold for the design of vaccines and therapeutics [4]–[7]. The ability to generate nanoscale materials that can specifically target and image sites of disease is an important goal in biomedicine. A variety of nanoparticle strategies have been developed for targeting and imaging *in vivo* including antibodies [8], dendrimers [9], liposomes [10], nanoshells [11], quantum dots [12], and viruses [13],[14]. Viruses are particularly suited for these applications because they are naturally designed for efficient circulation and specific ligand-binding and cellular internalization. Recently interest has turned toward self-assembling plant viruses, bacteriophages [4], and protein cage [15] architectures that can be adapted for *in vivo* targeting purposes without the pathogenic properties of animal viruses. However these viruses must generally be tailored to recognize their targets using specific ligands.

As a nanoparticle, CPMV is a robust biomaterial that is systemically bioavailable through both oral and intravenous inoculation [16]. These properties have been integral to its use as a vaccine platform [17]–[19]. CPMV has also been studied for materials applications such as multilayer assembly and chemical scaffolds [20]. Recent studies have also shown that CPMV can be chemically modified with specific ligands to achieve tumor-specific targeting [14]. Although the host range for CPMV replication is restricted to plants, interestingly the unmodified CPMV capsid also naturally interacts with mammalian cells. Intravital imaging studies using fluorescently-labeled CPMV particles yielded high-resolution images of normal and tumor vasculature *in vivo* [13]. These imaging studies showed that CPMV particles were readily internalized in mouse and chick endothelial cells following intravenous administration in living embryos, and this internalization produced high-resolution images of vasculature in real-time using epifluorescence microscopy [13]. Tumor neovasculature in particular was labeled very strongly by CPMV, and differential internalization by arterial and venous vessels was also observed, however the mechanism of uptake was unknown [13].

We subsequently determined that CPMV binding is mediated by a specific interaction between CPMV and a surface-exposed, non-glycosylated 54 kD binding protein that is present on a variety of mammalian cells including human umbilical vein endothelial cells (HUVEC) [21]. Since the interaction between CPMV and the 54 kD protein correlated with such high-resolution intravital vascular images, we reasoned that identifying the 54 kD CPMV attachment protein would potentially reveal a useful endothelial marker for vascular imaging. We also hypothesized that understanding the mechanism of CPMV attachment to mammalian cells would provide important information regarding the relationships between plant and animal picornaviruses. Thus the aim of this study was to identify and characterize the 54 kD CPMV binding protein (CPMV-BP) using proteomics, biochemical assays, flow cytometry, and fluorescence confocal microscopy.

## Results

**Figure 1:**
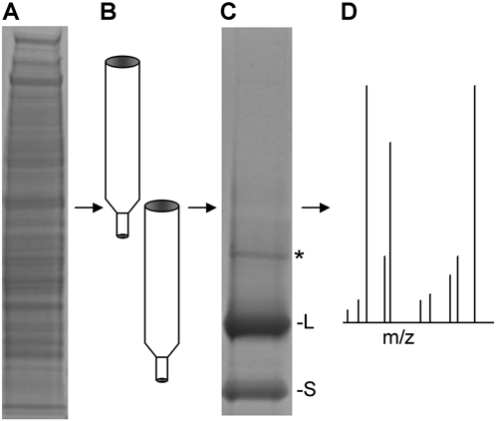
Strategy for identification of CPMV binding protein (CPMV-BP). (A) Plasma membrane enriched fractions isolated from BalbCl7 cells (run on SDS-PAGE gel and visualized with SimplyBlue). (B) Enriched plasma membranes were purified over concanavalin A- (to exclude glycoproteins) and CPMV- sepharose columns. (C) CPMV-sepharose bound material separated on SDS-PAGE gel (visualized with SimplyBlue). * = CPMV-BP, L = CPMV large subunit, S = CPMV small subunit. (D) CPMV-BP band (*) was excised from gel and identified through LC-MS/MS.

To identify the 54 kD CPMV-BP, a proteomics study was performed using liquid chromatography and tandem mass spectrometry (LC-MS/MS). The 54 kD protein is found in the plasma membrane-enriched fraction of cells, lacks N- and O-glycosylation, and was identified by its ability to bind directly to CPMV particles using a Virus Overlay Protein Blot Assay (VOPBA) [21]. The VOPBA technique has identified many high-affinity virus receptors including those for coronaviruses [22],[23], adenoviruses [24], and arenaviruses [25]. Mass spectrometry analysis focused first on enriched plasma membrane proteins that co-migrated with the 54 kD band on SDS-PAGE; this resulted in identification of 68 individual proteins (Table 1). Surface biotinylation of cells, followed by isolation of enriched plasma membranes and streptavidin-sepharose purification of biotinylated proteins, separation on SDS-PAGE and VOPBA, yielded a sample in the 54 kD range that was also analyzed by LC-MS/MS and yielded 7 proteins (Table 1). The third approach used the enriched plasma membrane fraction as starting material (Figure 1A) followed by sequential column chromatography (Figure 1B). Here the membrane fraction was first run over a concanavalin A-sepharose column to remove glycoproteins, and the flow-through fraction was bound to an affinity matrix that was generated when surface lysine residues on purified CPMV particles were directly conjugated to *N*-hydroxysuccinimidyl ester-sepharose (CPMV-sepharose). The CPMV-bound sample was washed several times, and then CPMV-sepharose beads were pelleted by centrifugation and bound proteins separated on SDS-PAGE (Figure 1C). Three bands were easily visible by SimplyBlue (Invitrogen) staining, corresponding to the 54 kD CPMV-BP, the 42 kD large capsid subunit of CPMV, and the 24 kD small CPMV capsid subunit. The 54 kD band was excised from the gel, digested with trypsin, and analyzed by LC-MS/MS using nano-electrospray on a linear ion trap mass spectrometer (Figure 1D). In this analysis two proteins were positively identified: vimentin and keratin. Keratin is a common laboratory contaminant that is isobaric with vimentin. Vimentin was also identified in all the preceding mass spectrometry analyses (Table 1). A complete listing of all proteins identified in each screen can be seen in Table S1.

**Table 1:** Summary of proteomic analyses used for identifying CPMV-BP.

Proteomic Analysis	Description	Number of Proteins Identified by >2 Peptides at 95% Confidence Level	Peptides Identified from Vimentin	% Sequence Coverage	Confidence Level of Vimentin ID
**1**	Enriched plasma membrane proteins that co-migrated with 54 kD band	68	28	56%	100%
**2**	Surface proteins isolated through biotinylation and co-migrated with 54 kD band	7	14	33%	100%
**3**	Enriched plasma membrane proteins which were purified through sequential column chromatography (Fig. 1) and co-migrated with 54 kD band	2	2	7%	100%

Vimentin is a type III intermediate filament and a major component of the cytoskeleton. Expressed in cells of mesenchymal origin, vimentin plays a key role in intracellular dynamics and architecture [26]. Vimentin encodes head, rod, and tail domains, and these domains are identified based on sequence and function [26]. Although it has long been considered a cytosolic protein, surface-expressed forms of vimentin have recently been discovered on several cell types including apoptotic neutrophils and T cells [27],[28], activated macrophages [29], platelets [30], vascular endothelial cells [31], brain microvascular endothelial cells [32], Sezary T cells [33], and skeletal muscle cells [32]. The mechanism by which vimentin reaches the cell surface, which domains are exposed, and its function at the surface, remain unknown.

**Figure 2:**
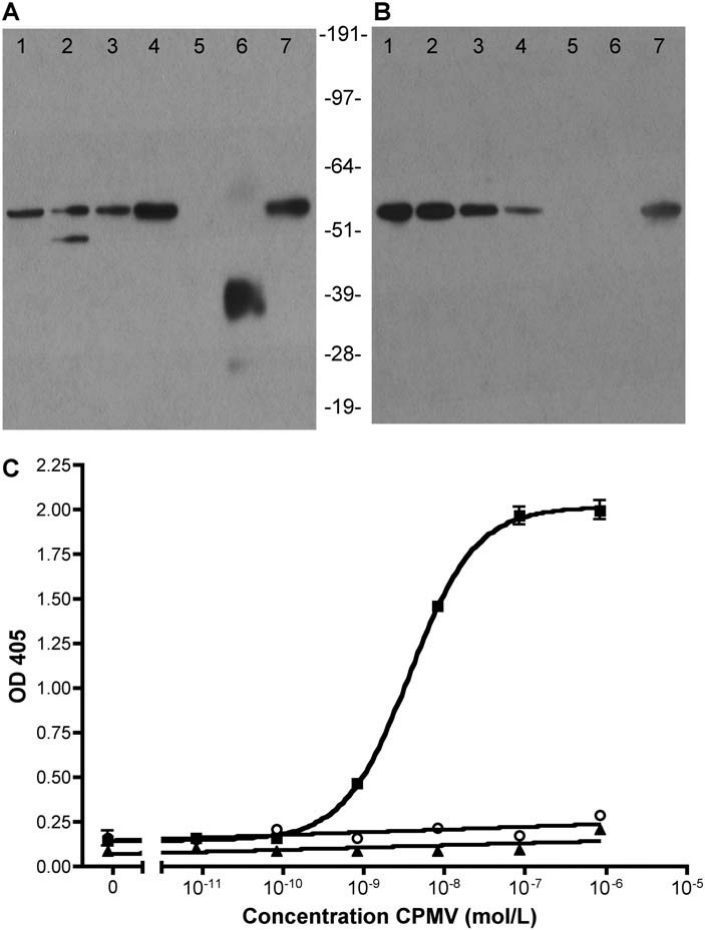
CPMV virions bind specifically to vimentin. (A) VOPBA and (B) α-vimentin western blot of enriched plasma membrane isolates of the following samples 1 = HUVEC, 2 = HeLa, 3 = KB, 4 = MFT-6 vim^+/+^, 5 = MFT-16 vim^−/−^; 6 = purified CPMV particles, 7 = recombinant vimentin protein. (C) Proteins were immobilized on ELISA plate, incubated with CPMV in varying molar concentrations and probed for CPMV attachment through antibody detection. Immobilized proteins were vimentin (squares), BSA (triangles) and no protein (open circles). Bars represent mean+/−s.d. of triplicate samples.

To evaluate whether surface-expressed or membrane associated vimentin interacted specifically with CPMV, VOPBA (Figure 2A) and western blotting (Figure 2B) were used to probe the interaction. Since vascular endothelial cells are known to internalize CPMV *in vivo* [13], enriched plasma membrane proteins isolated from HUVEC were used as a positive control (lane 1), along with HeLa and KB tumor cells (lanes 2 and 3) [21]. All cells contained the 54 kD band when probed with CPMV particles by VOPBA (Figure 2A). Significant signal could be observed even when incubating the virus with membrane for as little as 5 minutes. Mouse embryo fibroblasts derived from knockout mice lacking the vimentin gene (vim^−/−^, MFT-16; lane 5) [34],[35] were negative for the 54 kD CPMV-interacting band, while membranes isolated from control vim^+/+^ fibroblasts (MFT-6; lane 4) contained the 54 kD protein and bound CPMV by VOPBA. CPMV also bound to purified recombinant vimentin protein (lane 7), which migrated at the expected molecular weight. Expression of vimentin in the cell lines as detected by western blot using anti-vimentin antibodies (Figure 2B) correlated directly with binding of CPMV in VOPBA. CPMV capsid proteins were also included on the gels (Figure 2A, lane 6) as a positive control in the VOPBA for detection by CPMV-specific polyclonal antibodies. As expected CPMV capsid proteins did not react with anti-vimentin antibodies in the western (Figure 2B, lane 6; in addition, a loading control for the VOPBA and western blot samples is provided in Figure S1). Together these results demonstrate that vimentin is present in the enriched plasma membrane fraction of human cells and binds directly to CPMV.

The specificity of the vimentin-CPMV interaction was further probed in ELISA format. Purified protein (vimentin, BSA, or no protein control) was coated on ELISA plate wells overnight, and then incubated with varying concentrations of purified CPMV particles for one hour, followed by anti-CPMV polyclonal antibody and an alkaline-phosphatase conjugated secondary antibody (Figure 2C). CPMV bound specifically to vimentin immobilized on the plates at an EC_50_ of 3.72 nM of CPMV. Binding affinities that have been established for other picornavirus-receptor interactions range from 100 nM to 10 µM [36]–[38]. It is important to note that the 3.72 nM value does not represent the K_D_ of the CPMV-vimentin interaction, because ELISA assay does not rule out the role of avidity in binding. Nevertheless, this experiment further demonstrates a direct and specific interaction between CPMV and vimentin. CPMV binding could also be competed by vimentin-specific antibodies (Figure S2).

**Figure 3:**
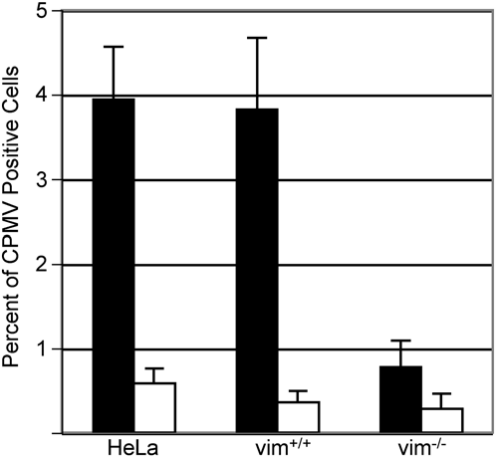
Cell-surface expression of vimentin promotes interaction with CPMV. Flow cytometry of surface vimentin-expressing cells HeLa and MFT-6 vim^+/+^, and vimentin negative MFT-16 vim^−/−^ cell types incubated with (black bars) and without (white bars) 10^5^ CPMV particles per cell for 30 minutes at 37°C in respective growth media. Bars represent mean+/−s.d. of triplicate samples.

The capacity of cell surface-expressed vimentin to mediate interactions with CPMV was further probed by flow cytometry at 37°C. Cells were incubated with CPMV for 30 minutes, fixed, and the amount of associated CPMV was detected with a CPMV-specific polyclonal antibody. HeLa and MFT-6 vim^+/+^ cells were capable of binding or internalizing CPMV, while MFT-16 vim^−/−^ cells could not (Figure 3). Since CPMV does not replicate in mammalian cells, the virus detected represents input virus particles only. In order to determine whether complementing vimentin expression in MFT-16 vim^−/−^ cells would increase the CPMV interaction, these cells were transfected with vimentin cDNA. Although MFT-16 vim^−/−^ cells had low transfection efficiencies (ranging from 0.1 to 8.0% of cells using a GFP-reporter plasmid, not shown), in the cell population that was transfected with vimentin, a statistically significant (p = 0.036) increase in CPMV binding or uptake was observed when compared to mock-transfected MFT-16 cells. On average 1.21% of vimentin-transfected MFT-16 vim^−/−^ cells were CPMV-positive (data not shown). For comparison, under identical conditions 5.36% of mock-transfected HeLa cells were CPMV positive (data not shown). Together these results demonstrate that the lack of CPMV binding observed in vimentin-null cells can be complemented by vimentin expression.

To further test the specificity of CPMV for surface-expressed vimentin in cells, antibody-blocking studies were performed. HeLa cells were incubated with various vimentin domain-specific antibodies at varying concentrations for 30 minutes, followed by a 30-minute incubation with CPMV under the identical growth conditions, and CPMV detected as described before. Anti-vimentin monoclonal antibody V9 that targets the tail domain was best at reducing CPMV binding, however, all antibodies did provide some blocking of CPMV binding or entry (Table 2). This inhibition is not complete, suggesting that either the monoclonal or polyclonal antibodies do not bind directly to the CPMV-interacting domains, that additional cell-surface interactions participate in CPMV binding, or that particular domains of vimentin may be more surface-exposed than other domains.

**Table 2:** Ability of anti-vimentin antibodies to block CPMV interaction with HeLa cells *in vitro* (mean±s.d.).

Antibody	Polyclonal or Monoclonal	Vimentin Epitope Region	Percent of CPMV Uptake Blocked at 1∶500 dilution	Percent of CPMV Uptake Blocked at 1∶100 dilution	Percent of CPMV Uptake Blocked at 1∶50 dilution
**H84**	Polyclonal	Head	−7.8±29	13±5.6	12±15
**3B4**	Monoclonal	Rod	−1.1±36	33±20	36±14
**V4630**	Polyclonal	Rod, Tail	27±19	38±11	42±27
**V9**	Monoclonal	Tail	2.8±8.0	48±10	60±7.9

**Figure 4:**
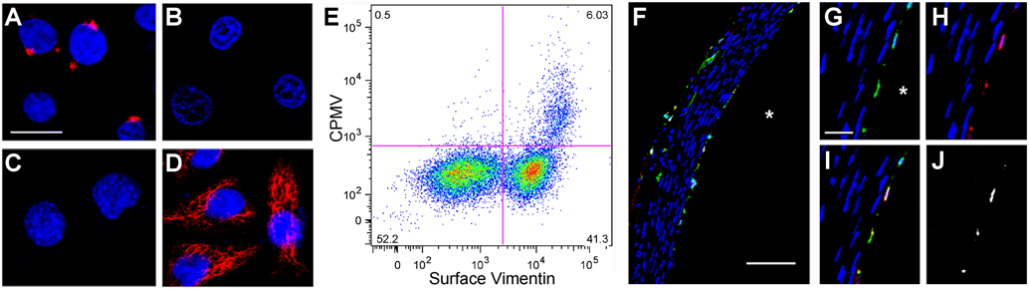
Correlation of vimentin surface expression and CPMV binding in cells and vasculature. (A) Surface vimentin expression on HeLa cells detected by V9 IgG1 antibody. Bar = 10 µm. (B) IgG1 isotype control. (C) Secondary antibody only. (D) Cytosolic vimentin expression in permeabilized HeLa cells detected by V9 IgG1 antibody. (E) Surface vimentin expression on HeLa cells (X-axis), and ability to interact with CPMV (Y-axis) were analyzed by flow cytometry. (F–J) CPMV and surface vimentin co-localize on the lumenal surface of rat aorta. Freshly isolated rat aorta was incubated with CPMV and vimentin antibodies *ex vivo*, and then 10 µm cryosections made and stained with secondary antibodies for confocal microscopy. F: co-localization of CPMV and vimentin on aortic endothelium. * = aorta lumen. G–J: individual detection and co-localization of CPMV and vimentin. Green = vimentin (panel F, G, I); red = CPMV (panel F, H, I); co-localization of CPMV and vimentin signal = white (F, I, J), where J shows only co-localized signal. Blue = DAPI stain for nuclei. Bar = 100 µm (F) and 25 µm (G) respectively.

To further correlate CPMV uptake with surface-vimentin expression in cell culture, HeLa cells were examined for surface vimentin by confocal microscopy and flow cytometry. Surface vimentin staining (Figure 4A) was shown to be markedly distinct from controls (Figure 4B and 4C) and internal vimentin staining (Figure 4D). The surface vimentin expression pattern observed in HeLa cells is virtually identical to confocal observation of surface vimentin expression previously reported in macrophages [29]. Fixation and staining procedures to verify cell surface and cytosolic staining were verified through the ability to detect β-COP, an intra-Golgi transport marker (Figure S3). Approximately 50% of HeLa cells exhibited surface vimentin expression by confocal microscopy, and surface vimentin expression was also quantified by flow cytometry when HeLa cells were stained using an anti-vimentin antibody (Figure S4). These data correlated with CPMV binding or uptake by flow cytometry (Figure 4E). CPMV-positive cells were observed within the surface-vimentin-expressing population in FACS (41.3% of the total population), although not all of these cells interacted with significant quantities of CPMV. The apparent CPMV binding of a few cells expressing surface vimentin at low levels was attributed to background staining (Figure S5).

Since we do not fully understand the expression, display, function, and availability of surface vimentin or its CPMV binding epitope it is difficult to hypothesize why some surface vimentin expressing cells are not also positive for CPMV. Nevertheless it is clear that surface vimentin is a prerequisite for CPMV interactions at the cell surface.

The interaction between CPMV and surface vimentin was then examined in animals. We first confirmed our previous results that CPMV interacts specifically with endothelial cells in vivo by staining with the CD31 marker. To this end, a mouse was intravenously injected with CPMV-A555, and after one hour the mouse was anesthetized, the aorta perfused with PBS and removed. The freshly isolated aorta was then incubated with antibodies recognizing the endothelial marker CD31/PECAM *ex vivo*, and under fluorescent microscopic observation there was strong co-localization between the CD31-expressing endothelial cells and CPMV (Figure S6).

Next, studies to co-localize CPMV and surface vimentin were performed. It had previously been suggested that vimentin is expressed on the lumenal surface of vascular endothelial cells, in particular by identification of the endothelial-specific PAL-E antibody (although the specificity of this antibody for vimentin is controversial) [31]. Because the expression of murine vimentin is not efficiently recognized by the V9 mAb, we used rats for our studies to co-localize vimentin and CPMV in vivo. In order to focus on surface vimentin displayed on the lumenal surface of vascular endothelium rather than cytosolic vimentin, cylinder-shaped rat aorta segments were excised, and prior to sectioning were incubated with CPMV-A555 or V9 mAb, washed, and then embedded in OCT medium and 10 µm cryosections prepared. The sections were then stained with a secondary anti-mouse antibody. The expression of vimentin was observed on the lumenal surface of aortic endothelium, and co-localized with CPMV binding (Figure 4F–J, and Figure S7). The colocalization of CPMV and vimentin in vessels correlated with the observed vascular endothelial uptake of CPMV *in vivo* (Figure S6 and [13]). Specificity of the staining procedure was also confirmed (Figure S8). There was no CPMV colocalization outside of the endothelial cell marker (Figure S6), or outside the surface vimentin expressing cells (Figure 4F–J). Taken together, the CPMV colocalization with vascular endothelium, the CPMV interaction with living vasculature shown previously through intravital imaging [13], and our current results indicating that surface vimentin facilitates this interaction, illustrates that endothelial cell targeting of CPMV is mediated via vimentin.

## Discussion

Together these studies identify the 54 kD protein that mediates binding of CPMV in mammalian cells as surface vimentin. These results demonstrate that CPMV is a useful nanoparticle probe for examining the expression of surface vimentin on endothelial cells and circulating cells *in vivo*. The ability of CPMV to efficiently visualize tumor neovasculature and differentiate arterial from venous tissues [13] may now be attributed to upregulation of surface vimentin. Upregulation of cytosolic vimentin has long been associated with tumor progression and metastasis during the epithelial-mesenchymal transition (EMT) [39], however our findings suggest that increased surface vimentin is also a key feature of tumor endothelium as evident by ability of CPMV to preferentially image these areas [13] and may signal a role for surface vimentin in tumor metastasis or invasion, in addition to cellular adhesion and stress. The use of CPMV as a natural endothelial probe may also extend into the investigation of other vascular diseases such as atherosclerosis. Finally, the CPMV-vimentin interaction may provide a tool for understanding the display and internalization of surface-expressed vimentin, the mechanism and function of which is currently unknown.

It is also not clear whether the CPMV-vimentin interaction is important for virion movement in its host plant species. CPMV is not known to be dependent on cellular receptors for cell-cell spread; rather like many plant viruses CPMV encodes a movement protein (MP) that mediates movement of virus particles within leaf tissue via the plasmodesmata [40],[41]. The mechanism of CPMV loading and unloading from the plant host's vascular elements is not understood [42]. Access to plant vascular tissues may be mediated by direct virus capsid-cellular interactions independent of MP, and intermediate filament-like proteins may play a role in the vascular tropism of CPMV in plants.

Interestingly the animal picornavirus TMEV has also been shown to interact directly with vimentin using a similar VOPBA strategy with isolated enriched plasma membrane proteins [43]. Coupled with our findings, this further strengthens the link between animal and plant picornaviruses not only structurally and genetically, but with regard to attachment mechanisms as well. The original characterization of CPMV bioavailability was performed following oral administration of virus or infected leaves, whereby virus was subsequently found in the blood circulation [16]. The stability of CPMV in the gastrointestinal tract and its subsequent systemic biodistribution provides an opportunity for interactions between CPMV and mammalian cells following ingestion [16]. While there is no evidence for CPMV replication in mammalian or avian cells, the conserved interaction with vimentin, coupled with the conserved capsid structures of the plant, insect and animal picornaviruses, supports the hypothesis that the picornavirus superfamily of viruses evolved from a common ancestor. The identification of conserved mechanisms of attachment and entry also point to a possible mode of cross-kingdom transmission.

Other roles for vimentin during the picornavirus replication cycle include reorganization of cytosolic vimentin into cages that enclose autophagic vesicles at intracellular replication centers by TMEV and poliovirus [43]–[45]. The picornavirus encephalomyocarditis virus (EMCV) further induces an autoimmune response against vimentin after infection [46].

In addition to picornaviruses, several other pathogens use vimentin as a component of the cellular attachment mechanism, suggesting a conserved role for surface vimentin as a more general attachment factor for pathogen entry. These include mammalian porcine reproductive and respiratory syndrome virus (PRRSV), which uses surface vimentin for cellular entry [47]. Bacteria such as *Escherichia coli* also interact with surface vimentin to mediate cellular attachment via the invasion factor *IbeA* [48]. Finally, upregulation of surface vimentin on injured skeletal muscle cells was recently shown to be a ligand for attachment of group A streptococci (GAS) and was associated with streptococcal toxic shock syndrome [32]. Together these studies highlight an increasingly important role for surface vimentin as a conserved component of pathogen attachment and internalization pathways, and suggest that disruption of these interactions may serve as broad-spectrum antimicrobial strategies.

## Materials and Methods

### Cell Culture

HeLa cells were grown and maintained in DMEM media supplemented with 7% heat-inactivated fetal bovine serum (ΔFBS), 50 units/mL penicillin, 50 units/mL streptomycin, and 2 mM L-glutamine. Murine Balb Cl 7 cells were grown and maintained in MEM media supplemented with 7% ΔFBS, 50 units/mL penicillin, 50 units/mL streptomycin, and 2 mM L-glutamine. HUVEC cells were grown and maintained using Endothelial Cell Growth Media Bulletkit (Cambrex). KB cells were grown and maintained in MEM media supplemented with 10% ΔFBS, 50 units/mL penicillin, 50 units/mL streptomycin, and 2 mM L-glutamine. MFT-6 and MFT-16 cells (a generous gift from Dr. Robert Evans, University of Colorado Health Sciences Center) were grown and maintained in 1∶1 DMEM and F-12 HAMS medias supplemented with 50 units/mL penicillin, 50 units/mL streptomycin, 2 mM L-glutamine, and 5% and 9% ΔFBS respectively. All cells were grown at 37°C in 5% CO_2_/95% air humidified atmosphere.

### Viruses

CPMV was grown, isolated, and when needed fluorescently labeled with AlexaFluor 488 or AlexaFluor 555 carboxylic acid, succinimidyl ester (Invitrogen) as described previously [13]. Labeled virus was calculated to have 65 AlexaFluor 488 molecules per virion or 55 AlexaFluor 555 per virion.

### Antibodies

Rabbit polyclonal anti-CPMV antibody was generated as previously described [21]. Antibodies against vimentin were rabbit polyclonal H-84 (Santa Cruz Biotechnology), mouse monoclonal 3B4 (Chemicon), whole goat antiserum V 4630 (Sigma) and mouse monoclonal IgG1 V9 (Sigma). Primary rat monoclonal anti-PECAM (CD31) was purchased from BD Biosciences. Secondary antibodies were donkey anti-goat IgG-HRP (Santa Cruz Biotechnology), goat anti-rabbit IgG-HRP (Pierce), goat anti-mouse Alexafluor 488 conjugated antibody (Invitrogen), goat anti-rabbit Alexafluor 488 conjugated antibody (Invitrogen), goat anti-mouse Alexafluor 647 conjugated antibody (Invitrogen) and goat anti-rat Alexafluor 488 conjugated antibody (Invitrogen). IgG1 isotype control was purchased from BD Biosciences and donkey biotinylated anti-rabbit IgG antibody from Amersham Biosciences.

### Enriched Plasma Membrane Protein Isolations

Cell lines were propagated and enriched plasma membranes isolated and stored in 10 mM Tris/HCl, pH 8.0, 10 µg/mL aprotinin and leupeptin (Roche), and 0.5% n-octyl-β-D-glycopyranoside (Sigma) as described previously [21]. The surface membrane-impermeable biotinylation of cells surface proteins and isolation was performed using the Cell Surface Protein Isolation Kit (Pierce) as directed by the manufacturer.

### CPMV-Sepharose Beads

To create CPMV-sepharose beads, 100 µl of NHS-activated Sepharose 4 Fast Flow (Amersham Biosciences) beads were washed with 1 mL of 1 mM HCl for 5 minutes at 4°C. HCl solution was removed and 18 mL of 0.1 M KPO_4_, pH 7.0 was added. 2 mL of 15 mg/mL CPMV in 0.1 M KPO_4_, pH 7.0 was added to NHS-activated sepharose already in solution. This mixture was rotated slowly on a LabQuake rotator (Cardinal Health) at room temperature for 2 hours. The mixture was centrifuged for 2 minutes at 100 g to pellet beads and remove excess solution. 20 mL of 0.1 M Tris/HCl, pH 8.0 was then added and put on slow rotation using the LabQuake rotator overnight at room temperature to hydrolyze unreacted NHS-esters. The resultant CPMV-sepharose beads were then extensively washed with 0.1 M KPO_4_, pH 7.0.

### Western and VOPBAs

10 µg of enriched plasma membrane protein isolates, from respective cell lines, were run on 4–12% Bis-Tris 1.0 mm NuPAGE gel (Invitrogen) unless otherwise specified. Proteins samples were then transferred electrophoretically to Immobilon-P transfer membranes (Millipore). Transfer membranes were then blocked overnight with 5% w/v milk solution. The membranes were then washed 4 times for 5 minutes each with wash buffer consisting of PBS with 0.2% Triton X-100 (Sigma). All antibodies and viral suspensions were diluted in wash buffer. For western blotting, samples were subject to one hour incubation with anti-vimentin whole goat antiserum V 4630, washed 4 times with wash buffer for 5 minutes each, then incubated one hour with donkey anti-goat IgG-HRP, washed 4 times with wash buffer for 5 minutes each, visualized with chemiluminescence detection (SuperSignal; Pierce) and exposed to CL-XPosure film (Pierce). For VOPBA, samples were subject to one hour incubation with 10 µg/mL CPMV in 1% milk solution with 5% glycerol, washed 4 times with wash buffer for 5 minutes each, then subject to one hour incubation with anti-CPMV polyclonal antibody, washed 4 times with wash buffer for 5 minutes each, then incubated one hour with goat anti-rabbit IgG-HRP, washed 4 times with wash buffer for 5 minutes each, visualized with chemiluminescence detection (SuperSignal; Pierce) and exposed to CL-Xpossure film (Pierce).

### Proteomic Analysis

#### Screen 1

Two 40 µg samples of Balb Cl 7 enriched plasma membrane isolate was separated on a single 4–12% Bis-Tris 1.5 mm NuPAGE gel. One sample was subjected to VOPBA analysis and the resultant CPMV reactive band seen on X-ray film was used to target and excise the similar region of the other sample.

#### Screen 2

Balb Cl 7 cell surface proteins were isolated using the Cell Surface Protein Isolation Kit (Pierce). Four T75 tissue culture flasks containing confluent monolayers of Balb Cl 7 were used for isolation. The consequential surface proteins were split into two samples, and separated on a single 4–12% Bis-Tris 1.5 mm NuPAGE gel. One sample was subjected to VOPBA analysis and the resultant CPMV reactive band seen on X-ray film was used to target and excise the similar region of the other sample.

#### Screen 3 (sequential column chromatography)

218 µl of 4.6 mg/mL Balb Cl 7 enriched plasma membrane isolate was mixed with 400 µl of 0.1 M KPO_4_, pH 7.0. This mixture was run over a 1 mL Concanavalin A Sepharose (Amersham Biosciences) column, and washed with 2 mL 0.1 M KPO_4_, pH 7.0. The flow through was then incubated with 100 µl of CPMV-sepharose beads for 10 minutes at room temperature. The CPMV-sepharose beads were then washed with 200 column volumes of 0.1 M KPO_4_, pH 7.0. CPMV-sepharose beads were drained, SDS-PAGE reducing buffer added, boiled at 95°C for ten minutes, split into two equal volumes, and each separated on single 4–12% Bis-Tris 1.5 mm NuPAGE gel. One sample was subject to VOPBA to identify capture of CPMV-BP, and the other visualized through SimpleBlue Stain (Invitrogen). CPMV-BP band was excised.

### Chromatography and Mass Spectrometry

The excised gel bands for proteomic analysis were treated with 10 mM dithiothrietol to reduce disulfide linkages. Alkylation was performed with 55 mM iodoacetamide (Sigma-Aldrich) before digestion with trypsin (Promega) over night at 37°C using an estimated (1∶30) enzyme to substrate ratio in 50 mM ammonium bicarbonate. The liquid chromatography separation was performed on a laser pulled 100 µm ID C_18_ column with a tip of <5 µm that is also used as a nanoelectrospray emitter. An Agilent 1100 HPLC system equipped with a nanopump was used to perform the gradient elution at a flow rate of 300 nL/min with 0.1% formic acid/acetonitrile as the mobile phases, from 5% to 35% acetonitrile in 100 minutes, then up to 90% acetonitrile for 15 minutes. The MS/MS analysis was performed on a LTQ linear ion trap mass spectrometer (Thermo Electron Corp.), as well as an Agilent LC/MSD Trap ion trap mass spectrometer. Data-dependent scanning was used to maximize the number of peptides sequenced in the highly complex mixture. This mode of operation uses preset criteria to select unique peptides on-the-fly for undergoing MS/MS. Over 10,000 MS/MS spectra were obtained during the runs. These were searched using MASCOT (Matrix Science, Limited) with the Sprot protein database. To improve searching efficiency, taxonomic category was limited to rodent proteins. Only peptides producing good quality fragmentation spectra and scoring higher than the threshold required for 95% confidence level for Mascot were used for protein identification. A protein identification was only validated if two or more peptides were identified with ion scores needed for 95% confidence level.

### ELISA

One µg of vimentin, BSA or no protein at all, was suspended in 150 µL 0.1 M KPO_4_ pH 7.0, was immobilized overnight in 96-well Immulon 2 HB plates (Thermo). During the immobilization, plates were kept at room temperature and in buffer humidified containers. The next morning the protein solutions were discard and wells blocked for 2 hours at room temperature with 300 µL of 3% milk solution in 0.1 M TBS pH 7.3 with 0.05% Tween 20. All washes were completed with 0.1 M TBS pH 7.3 with 0.05% Tween 20, all viral and antibody dilutions were made in 150 µL 0.1 M KPO_4_ pH 7.0 and all incubations took place at room temperature unless specifically stated. After blocking wells they were washed and appropriate molar concentrations of virus were added to each well. For “no protein” wells the same amount of virus was used as was used in the vimentin wells. Viral incubations lasted 1 hour, was followed by 3 washes and 1 hour incubation with rabbit anti-CPMV polyclonal antibody. This was followed by 3 washes and 1 hour incubation with donkey biotinylated anti-rabbit IgG antibody. Then this was followed by 3 washes and 1 hour incubation with streptavidin-alkaline phosphatase (Amersham Bioscience). Another 3 washes were completed and p-nitriphenyl phosphate (Sigma) was incubated for 20 minutes at 37°C or until the negative control started to barely change color. The reaction was stopped by addition of 2N NaOH for 10 minutes at room temperature. Signal was recorded at 405 nm on a VERSAmax tunable microplate reader (Molecular Devices). All experiments were repeated in triplicate with average and standard deviation reported.

### Flow Cytometry

To detect CPMV interactions cells were dissociated from growth flask using Hanks'-Based, Enzyme Free, Cell Dissociation Buffer (Invitrogen), counted and resuspended in their respective growth media. These cells were then aliquoted into 96-well V-bottom plates. Plates were spun to collect cells after each addition of virus, fixative, antibody or washing. Cells were then incubated with wildtype CPMV in a ratio of 1×10^5^ virions per cell (V/C) for 30 minutes at 37°C. Cell were washed three times with FACS buffer consisting of PBS, 1 mM EDTA, 25 mM HEPES and 1% FBS at pH 7.0. Cells were fixed for 15 minutes with 2% formaldehyde in PBS and then washed three times with FACS buffer. Cells were then washed once with FACS buffer containing 0.5% saponin (Sigma) also called permeablization buffer (PB). Cells were then incubated with rabbit polyclonal anti-CPMV antibody diluted in PB for one hour at room temperature and then washed three times with PB. Secondary goat anti-rabbit Alexafluor 488 conjugated antibody was diluted in PB and incubated with the cells for one hour at room temperature in the dark. Cells were washed a final three times with FACS buffer, fluorescence quantitated with a LSR-II Digital Flow Cytometer (BD Biosciences) and data analyzed used FlowJo software (Tree Star Inc.). For antibody blocking experiment after HeLa cells were aliquoted they were incubated with varying concentrations (1∶50, 1∶100, 1∶500 or no antibody) of H-84, 3B4, V4630 or V9 antibody for 30 minutes at 37°C, then addition of AlexaFluor 488 labeled virus for 30 minutes at 37°C and procedure continued as discussed above. For *in vitro* CPMV binding or internalization and surface vimentin staining flow cytometry the same procedure was used except: AlexaFluor 488 labeled virus was used, no PB was used and surface vimentin analyzed through use of V9 anti-vimentin monoclonal antibody in place of rabbit polyclonal anti-CPMV polyclonal antibody and secondary goat anti-rabbit Alexafluor 488 conjugated antibody replaced with goat anti-mouse Alexafluor 647 conjugated antibody. For surface vimentin staining in Figure S4 cells were fixed, not permeablized, and staining using V9 anti-vimentin or mouse IgG1 isotype control and goat anti-mouse Alexafluor 647 conjugated antibody as discussed above. For each experiment at least 10,000 events were collected and were repeated in at least triplicate with average and standard deviations calculated in Microsoft Excel and reported.

### MFT-16 (Vimentin −/−) Transfection

The night before transfection 500,000 cells were seeded in 2 mL of growth media in 6-well plates and grown overnight to 90–95% confluence at 37°C in a 5% CO_2_/95% air humidified atmosphere. For each well of cells 4 µg of pCMV-SPORT6-vimentin (Open Biosystems), a vimentin plasmid with CMV promoter, or no DNA (mock transfection) was diluted in 250 µl of transfection media (growth media without ΔFBS or antibiotics). For each well 10 µl of Transfectin (Biorad) was diluted in 250 µl of transfection media, and incubate at room temp for 5 minutes. The diluted DNA and Transfectin was combined, mixed gently, and incubated at room temperature for 20 minutes. Growth media was aspirated from cells, and cells washed twice with PBS. The combined DNA and Transfectin was added dropwise to the well of cells and returned to 37°C in a 5% CO_2_/95% air humidified atmosphere. After 4 to 6 hours DNA/Transfectin mixture was aspirated off cells, cells washed twice with PBS, and 2 mL of growth media added to cells. Cells were returned to 37°C in a 5% CO_2_/95% air humidified atmosphere for 24 hours. Cells were removed from wells using Hanks'-Based, Enzyme Free, Cell Dissociation Buffer, counted and 500,000 cells placed in wells of a V-bottom 96-well plate. In V-bottom wells cells were then incubated with wildtype CPMV at 5×10^5^ V/C or no virus for 2 hours. CPMV interaction was measured using the flow cytometry procedure listed above. Transfection efficiency varied per experiment ranging from 0.1 to 8% efficiency. Transfection efficiency was measured through permeablization of cells and identification of vimentin expression using V9 anti-vimentin monoclonal antibody and goat anti-mouse Alexafluor 647 conjugated antibody while using PB buffer in flow cytometry preparation listed above. All transfections were repeated in triplicate.

### Confocal Microscopy of HeLa Vimentin Expression

HeLa cells were seeded in a 12-well plate containing 12 mm sterile glass cover slips at 5×10^4^ cells/well and grown for 48 hours in RPMI1640 medium containing 10% ΔFBS, 50 units/mL penicillin, 50 units/mL streptomycin, and 2 mM L-glutamine at 37°C in a 5% CO_2_/95% air humidified atmosphere. On the day of the experiment, cells were fixed using 3% paraformaldehyde, 0.3% gluteraldeyde, 1 mM MgCl_2_ in PBS for 10 minutes. After 4 washes with PBS buffer, only the cells that were to be intracellularly stained, were permeabilized using 0.2% Triton X-100 in PBS for 2 minutes. Non-specific binding was blocked by incubating the cells in 5% goat serum in PBS for 1 hour. Incubations with either mouse monoclonal anti-vimentin V9 antibody, rabbit polyclonal anti-β-COP (Affinity Bioreagents) or purified mouse IgG1 isotype control were performed at room temperature for 45 minutes with gentle agitation. Unbound antibody was then removed by washing four times with PBS. Goat anti-mouse IgG AlexaFluor 555 conjugated antibody (Invitrogen) or goat anti-rabbit IgG AlexaFluor 488 conjugated antibody (Invitrogen) were added appropriately in 1% goat serum in PBS, and cells were gently agitated for a further 45 minutes. During the last five minutes of secondary antibody incubation, cell nuclei were stained by adding 100 µL of 4′,6-diamidino-2-phenylindole (DAPI 1∶1000 dilution in water). Cells were then washed 4 times using PBS and cover slips covered with cells were mounted on slides using Vecta Shield mounting medium (Vector Laboratories). Cells were imaged using a Bio-Rad (Zeiss) Radiance 2100 Rainbow laser scanning confocal microscope equipped with 60× oil-immersion objective.

### *Ex Vivo* Rat Aorta Study

Animals used in this study were Harlan Sprague-Dawley male rats obtained from Charles River Inc. Animals were used in compliance with Institutional Animal Care and Use Committee (IACUC) approved protocols. On the day of the experiment, rats were anesthetized and the aorta perfused with ice-cold PBS for 10 minutes via the left ventricle. The aorta was then removed by cutting off minor branching arteries and rinsed in ice-cold PBS to remove adhering blood components. Aorta transverse segments were obtained and incubations with either mouse monoclonal anti-vimentin V9 antibody, purified mouse IgG1 isotype control, CPMV labeled with Alexafluor 555 (20 µg), or a combination of V9 and labeled virus were performed at 4C for 1 hr in the dark. V9 antibody was diluted 1 to 40 in 2% natural goat serum, 1% BSA in PBS. Incubation with IgG1 isotype control was performed so that the same amount of antibody as for V9 was used. The segments were then washed 3 times with ice-cold PBS in the dark and embedded in OCT (Tissue-Tek). 10 µm sections were obtained using a Leica CM1850 cryostat, collected on glass slides and fixed in ice-cold 95% ethanol for 30 minutes at 4C. After rinsing the slides with PBS, goat anti-mouse Alexafluor 488 conjugated antibody was added for 1 hr in the dark. In the last ten minutes of secondary antibody incubation, nuclei were stained using 4′, 6-diamino-2-phenylindole (DAPI). Slides were then washed 4 times with PBS and mounted using Vecta Shield mounting medium. Aorta segments were imaged using a Bio-Rad (Zeiss) Radiance 2100 Rainbow laser scanning confocal microscope equipped with 60× oil-immersion objective.

### *In Vivo* Mouse Aorta Study

C57Bl/6J mice (rodent breeding colony, TSRI) were used in accordance with IACUC approved protocols. On the day of the experiment, mice were inoculated intravenously using 500 µg of CPMV labeled with Alexafluor 555. After 1 hour the mice were anesthetized and the aorta perfused with ice-cold PBS for 10 minutes via the left ventricle. The aorta was then removed by cutting off minor branching arteries and rinsed in ice-cold PBS to remove adhering blood components. Aorta transverse segments were obtained and embedded in OCT. 10 µm sections were obtained using a Leica CM1850 cryostat, collected on glass slides and fixed in ice-cold 95% ethanol for 30 minutes at 4C. After rinsing the slides with PBS, blocking was performed using 10% natural goat serum in PBS for 30 minutes. The sections were then incubated with PECAM (CD31) primary antibody in 5% goat serum in PBS. After 1 hour, slides were washed four times in PBS. Goat anti-rat Alexafluor 488 conjugated secondary antibody was then added in 5% goat serum in PBS. In the last ten minutes of secondary antibody incubation, nuclei were stained using 4′, 6-diamino-2-phenylindole (DAPI). Slides were then washed 4 times with PBS and mounted using Vecta Shield mounting medium (Vector Laboratories). Aorta segments were imaged using a Bio-Rad (Zeiss) Radiance 2100 Rainbow laser scanning confocal microscope equipped with 60× oil-immersion objective.

## Supporting Information

Figure S1 Loading control for samples in Figure 2A and 2B, CPMV VOPBA and anti-vimentin western blot of enriched plasma membrane isolates. Lanes: 1 = HUVEC, 2 = HeLa, 3 = KB, 4 = MFT6 vim+/+, 5 = MFT16 vim−/−, 6 = purified CPMV particles, 7 = recombinant vimentin protein. Molecular weight standards are noted at left.
(0.07 MB PDF)

Figure S2 Anti-vimentin monoclonal antibody V9 inhibits CPMV binding to vimentin in both VOPBA and in ELISA format. (A) Using the VOPBA format as shown in Figure 2A, HeLa cell membrane-enriched fractions were separated on SDS-PAGE and transferred to membranes as described in Materials and Methods. Membranes were cut into strips and separately were incubated for one hour with V9 antibody (5.0 µg/mL), mouse IgG1 isotype control (5.0 µg/ml), or no antibody, prior to a five minute incubation with CPMV, and the VOPBA procedure continued as described in Materials and Methods. (B) Using the ELISA format as shown in Figure 2C, 0.9 µg of purified vimentin protein were immobilized per well, then wells were incubated for two hours with a 3% milk solution to block nonspecific binding, wells were extensively washed then incubated for one hour with anti-vimentin V9 or mouse IgG1 isotype control antibody (ratio on x-axis indicates molar ratio of antibody to immobilized vimentin used). Again wells were extensively washed then incubated with a 2-fold molar excess of CPMV, and the ELISA procedure continued as previously described for detection of CPMV binding in Figure 2C. Bars represent mean+/−S.D. of duplicate samples. * indicates p-value<0.05.
(0.19 MB PDF)

Figure S3 Establishing specificity of surface and internal staining of HeLa cells using confocal microscopy. (A) Surface HeLa expression of vimentin. (B) Surface HeLa expression of beta-COP. (C) Internal HeLa expression of vimentin. (D) Internal HeLa expression of beta-COP. Bar = 25 µm.
(0.10 MB PDF)

Figure S4 Surface vimentin expression on HeLa cells analyzed by flow cytometry. Samples of HeLa cells were stained with secondary antibody only (red histogram), mouse IgG1 isotype control (blue histogram) or V9 anti-vimentin (IgG1, green histogram). Marker indicates percentage of total population of cells that expressed surface vimentin compared to 0.19% for isotype control.
(0.10 MB PDF)

Figure S5 Controls for flow cytometry analysis of CPMV binding and vimentin expression on HeLa cells. HeLa cells were subjected to one hour incubation with labeled CPMV under growth conditions or surface vimentin staining. (A) Cells only. (B) Secondary antibody only. (C) After one hour incubation with labeled CPMV. (D) Vimentin surface staining of HeLa cells.
(0.14 MB PDF)

Figure S6 CPMV and CD31/PECAM (an endothelial marker) co-localize on the lumenal surface of mouse aorta. An adult C57Bl6J mouse was intravenously inoculated with Alexafluor 555-labeled CPMV. After one hour the mouse was anesthetized, aorta perfused with PBS and removed. From the freshly isolated aorta segments 10 µm cryosections were prepared and then cryosections were stained with CD31/PECAM antibodies. Blue = nuclei (DAPI), green = CD31/PECAM (A and C), red = CPMV (B and C), white = colocalization (C and D), * = lumen, and scale bar = 25 µm.
(0.11 MB PDF)

Figure S7 Additional examples of CPMV and surface vimentin co-localization on the lumenal surface of rat aorta evaluated by confocal microscopy. (A–E) Freshly isolated rat aorta was incubated with CPMV and vimentin antibodies *ex vivo*, and 10 µm cryosections made. Blue = DAPI, green = vimentin, red = CPMV, white = colocalization, * = vessel lumen, and bar = 100 µm (A) and 25 µm (B) respectively.
(0.10 MB PDF)

Figure S8 Establishing specificity of CPMV and vimentin staining in rat aorta via fluorescence confocal microscopy. Rat aortic segments incubated *ex vivo* with labeled CPMV or vimentin-specific antibodies were cryosectioned and stained with secondary antibodies. (A) CPMV. (B) Monoclonal anti-vimentin antibody. (C) Monoclonal isotype control. Bar = 10 µm, * = lumen.
(0.09 MB PDF)

Table S1 Protein name, accession number, number of peptides identified, and sequence coverage of all proteins found in proteomic analysis 1–3.
(0.04 MB XLS)
