# Supplementary material for: Endothelial Targeting of Cowpea Mosaic Virus (CPMV) via Surface Vimentin
Source: PLoS Pathog. 2009 May 1;5(5):e1000417. doi: 10.1371/journal.ppat.1000417 (PMC2670497; doi:10.1371/journal.ppat.1000417)
Supplement: Figure S1 — Loading control for samples in Figure 2A and 2B, CPMV VOPBA and anti-vimentin western blot of enriched plasma membrane isolates. Lanes: 1 = HUVEC, 2 = HeLa, 3 = KB, 4 = MFT6 vim+/+, 5 = MFT16 vim−/−, 6 = purified CPMV particles, 7 = recombinant vimentin protein. Molecular weight standards are noted at left. (0.07 MB PDF) [file ppat.1000417.s001.pdf]

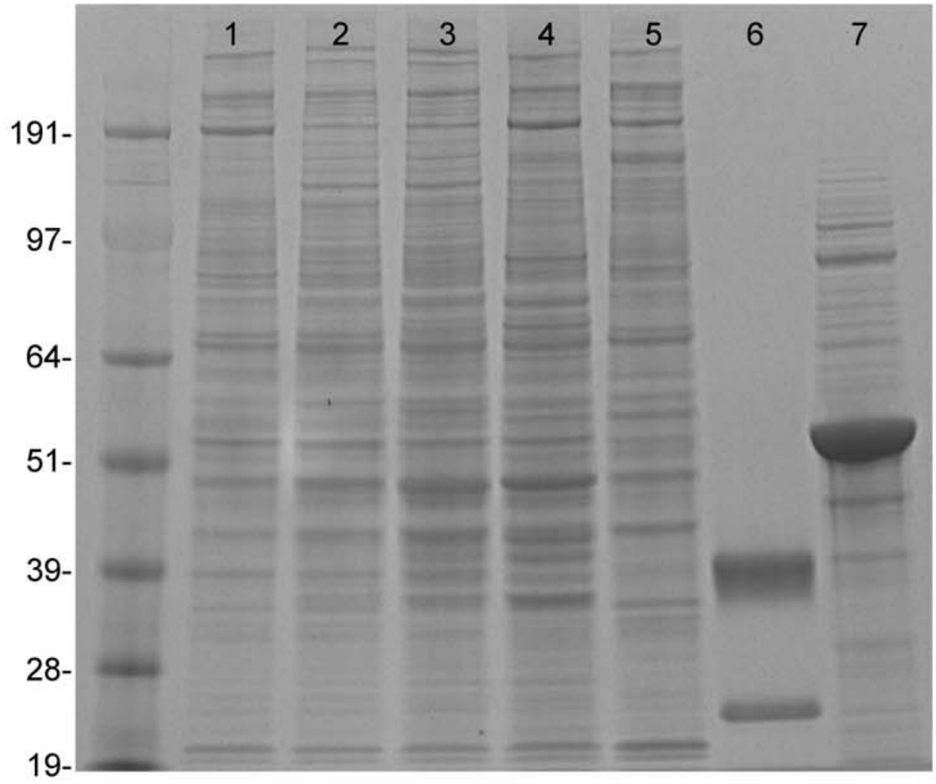

Figure S1: Loading Control for samples in Figure 2 A and B, CPMV VOPBA and anti-vimentin western blot of enriched plasma membrane isolates. Lanes: 1 = HUVEC, 2 = HeLa, 3 = KB, 4 = MFT-6 vim+/+, 5 = MFT-16 vim-/- ; 6 = purified CPMV particles, 7 = recombinant vimentin protein. Molecular weight standards are noted at left.
