# Supplementary material for: Endothelial Targeting of Cowpea Mosaic Virus (CPMV) via Surface Vimentin
Source: PLoS Pathog. 2009 May 1;5(5):e1000417. doi: 10.1371/journal.ppat.1000417 (PMC2670497; doi:10.1371/journal.ppat.1000417)
Supplement: Figure S2 — Anti-vimentin monoclonal antibody V9 inhibits CPMV binding to vimentin in both VOPBA and in ELISA format. (A) Using the VOPBA format as shown in Figure 2A, HeLa cell membrane-enriched fractions were separated on SDS-PAGE and transferred to membranes as described in Materials and Methods. Membranes were cut into strips and separately were incubated for one hour with V9 antibody (5.0 µg/mL), mouse IgG1 isotype control (5.0 µg/ml), or no antibody, prior to a five minute incubation with CPMV, and the VOPBA procedure continued as described in Materials and Methods. (B) Using the ELISA format as shown in Figure 2C, 0.9 µg of purified vimentin protein were immobilized per well, then wells were incubated for two hours with a 3% milk solution to block nonspecific binding, wells were extensively washed then incubated for one hour with anti-vimentin V9 or mouse IgG1 isotype control antibody (ratio on x-axis indicates molar ratio of antibody to immobilized vimentin used). Again wells were extensively washed then incubated with a 2-fold molar excess of CPMV, and the ELISA procedure continued as previously described for detection of CPMV binding in Figure 2C. Bars represent mean+/−S.D. of duplicate samples. * indicates p-value<0.05. (0.19 MB PDF) [file ppat.1000417.s002.pdf]

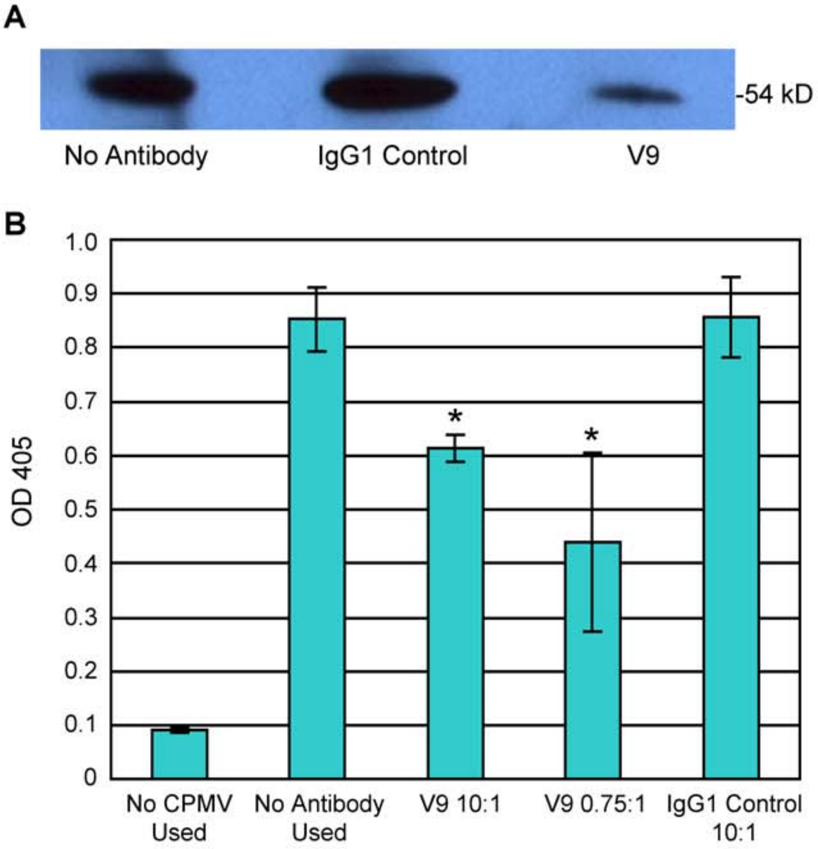

Figure S2: Anti-vimentin monoclonal antibody V9 inhibits CPMV binding to vimentin in both VOPBA and in ELISA format. (A) Using the VOPBA format as shown in Figure 2A, HeLa cell membrane-enriched fractions were separated on SDS-PAGE and transferred to membranes as described in Materials and Methods. Membranes were cut into strips and separately were incubated for one hour with V9 antibody (5.0 µg/mL), mouse IgG1 isotype control (5.0 µg/ml), or no antibody, prior to a five minute incubation with CPMV, and the VOPBA procedure continued as described in Materials and Methods. (B) Using the ELISA format as shown in Figure 2C, 0.9 µg of purified vimentin protein were immobilized per well, then wells were incubated for two hours with a 3% milk solution to block nonspecific binding, wells were extensively washed then incubated for one hour with anti-vimentin V9 or mouse IgG1 isotype control antibody (ratio on x-axis indicates molar ratio of antibody to immobilized vimentin used). Again wells were extensively washed then incubated with a 2-fold molar excess of CPMV, and the ELISA procedure continued as previously described for detection of CPMV binding in Figure 2C. Bars represent mean +/- S.D. of duplicate samples. * indicates p-value < 0.05.
