# Supplementary material for: Endothelial Targeting of Cowpea Mosaic Virus (CPMV) via Surface Vimentin
Source: PLoS Pathog. 2009 May 1;5(5):e1000417. doi: 10.1371/journal.ppat.1000417 (PMC2670497; doi:10.1371/journal.ppat.1000417)
Supplement: Figure S3 — Establishing specificity of surface and internal staining of HeLa cells using confocal microscopy. (A) Surface HeLa expression of vimentin. (B) Surface HeLa expression of beta-COP. (C) Internal HeLa expression of vimentin. (D) Internal HeLa expression of beta-COP. Bar = 25 µm. (0.10 MB PDF) [file ppat.1000417.s003.pdf]

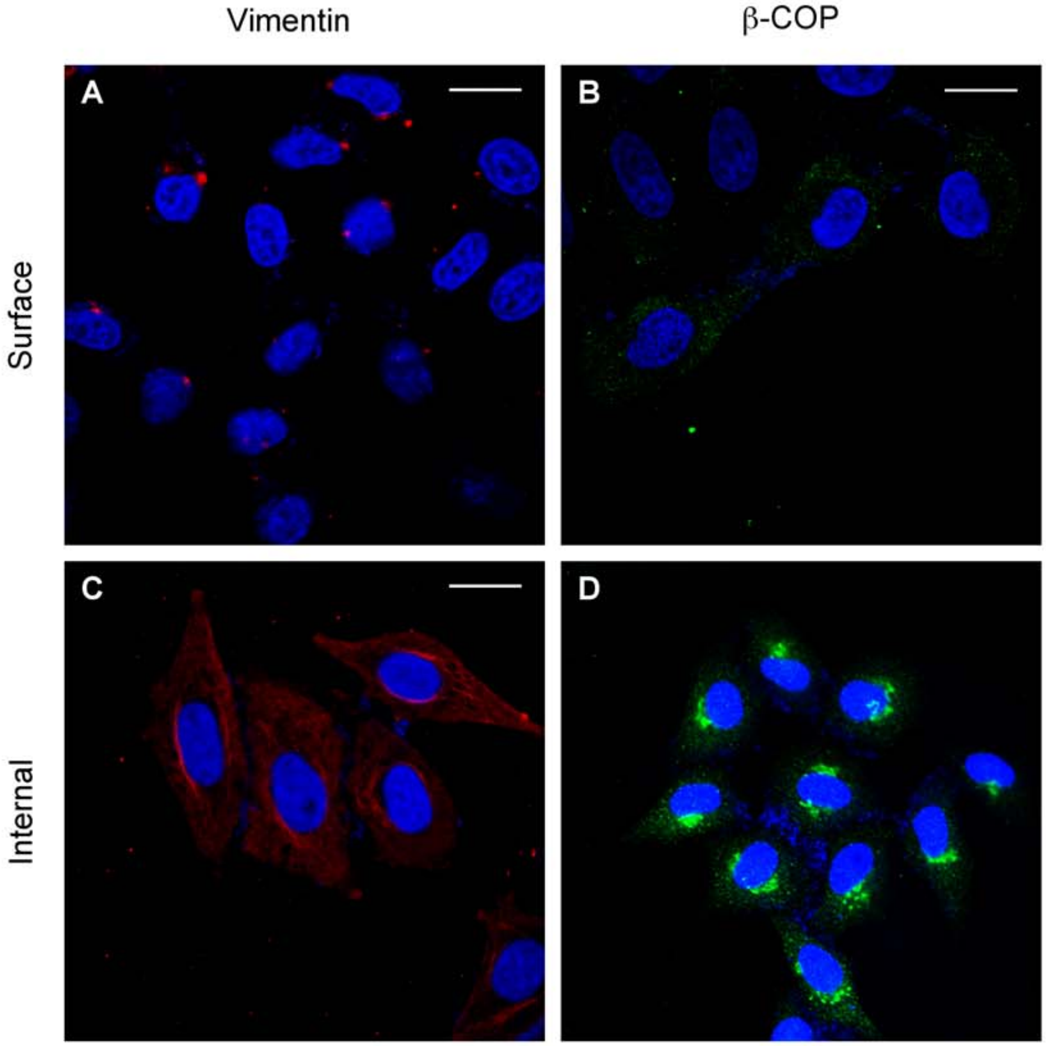


**Figure S3: Establishing specificity of surface and internal staining of HeLa cells using confocal microscopy.** (A) Surface HeLa expression of vimentin. (B) Surface HeLa expression of beta-COP. (C) Internal HeLa expression of vimentin. (D) Internal HeLa expression of beta-COP. Bar = 25μm.
