# Supplementary material for: Endothelial Targeting of Cowpea Mosaic Virus (CPMV) via Surface Vimentin
Source: PLoS Pathog. 2009 May 1;5(5):e1000417. doi: 10.1371/journal.ppat.1000417 (PMC2670497; doi:10.1371/journal.ppat.1000417)
Supplement: Figure S4 — Surface vimentin expression on HeLa cells analyzed by flow cytometry. Samples of HeLa cells were stained with secondary antibody only (red histogram), mouse IgG1 isotype control (blue histogram) or V9 anti-vimentin (IgG1, green histogram). Marker indicates percentage of total population of cells that expressed surface vimentin compared to 0.19% for isotype control. (0.10 MB PDF) [file ppat.1000417.s004.pdf]

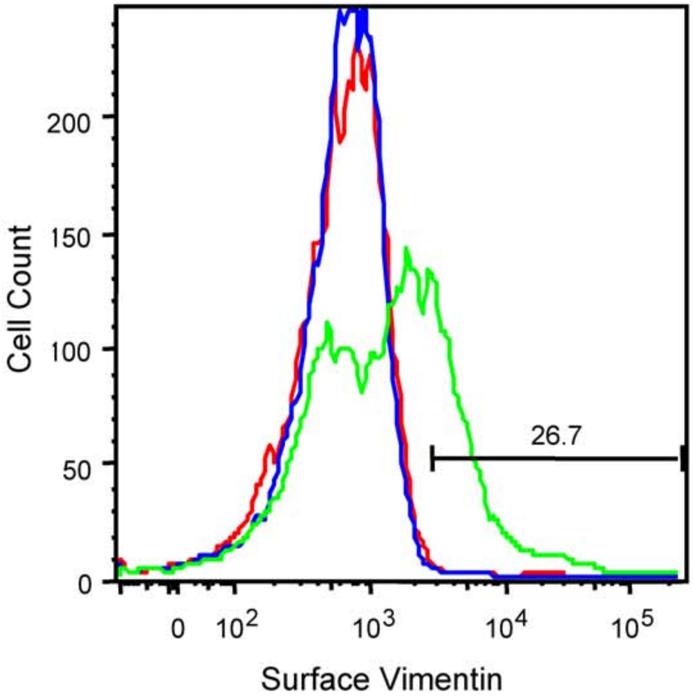


**Figure S4: Surface Vimentin Expression of HeLa Cells analyzed by flow cytometry.** Samples of HeLa cells were stained with secondary antibody only (red histogram), mouse isotype IgG1 control, (blue histogram), or V9 anti-vimentin (IgG1; green histogram). Marker indicates percent of the total population of cells that expressed surface vimentin compared to 0.19% for isotype control.
