# Supplementary material for: Endothelial Targeting of Cowpea Mosaic Virus (CPMV) via Surface Vimentin
Source: PLoS Pathog. 2009 May 1;5(5):e1000417. doi: 10.1371/journal.ppat.1000417 (PMC2670497; doi:10.1371/journal.ppat.1000417)
Supplement: Figure S5 — Controls for flow cytometry analysis of CPMV binding and vimentin expression on HeLa cells. HeLa cells were subjected to one hour incubation with labeled CPMV under growth conditions or surface vimentin staining. (A) Cells only. (B) Secondary antibody only. (C) After one hour incubation with labeled CPMV. (D) Vimentin surface staining of HeLa cells. (0.14 MB PDF) [file ppat.1000417.s005.pdf]

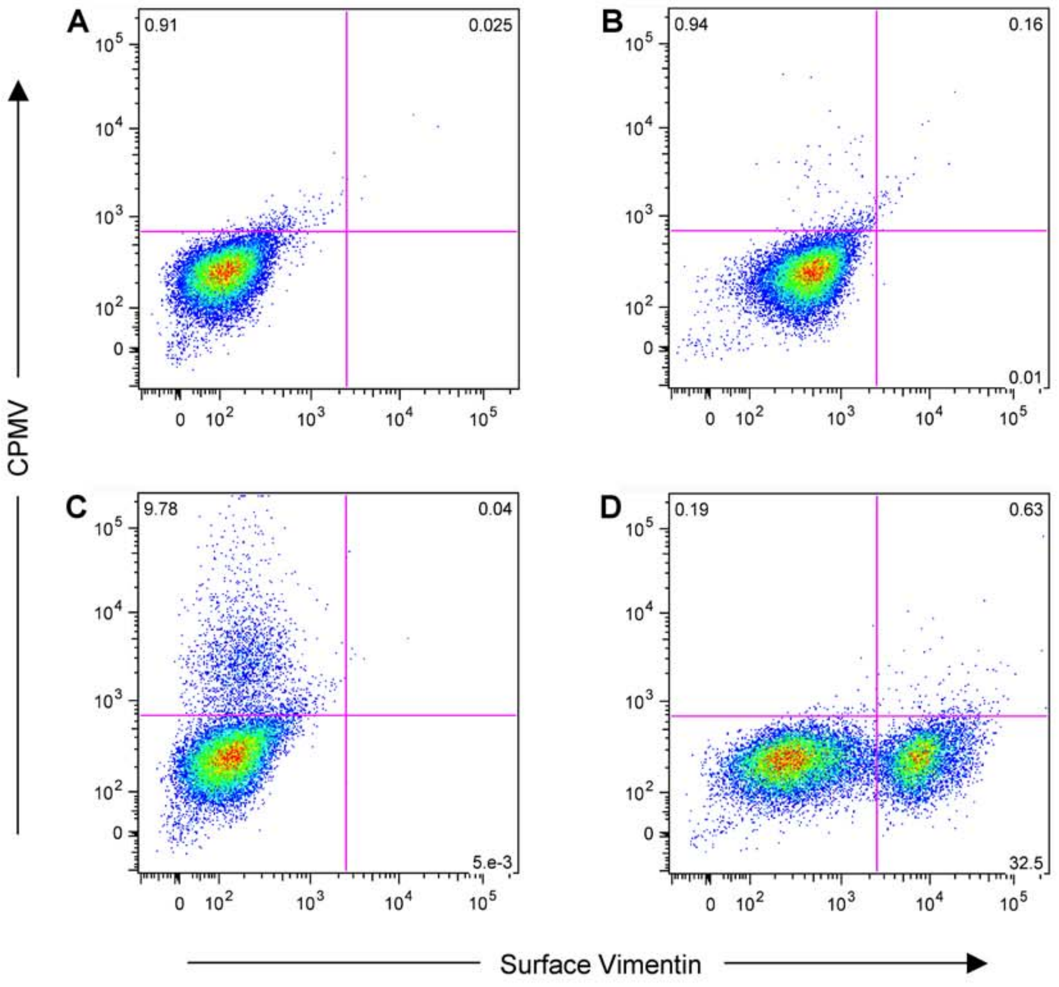

Figure S5: Controls for flow cytometry analysis of CPMV binding and vimentin expression on HeLa cells. HeLa cells were subjected to one hour incubation with labeled CPMV under growth conditions or surface vimentin staining. (A) Cells only. (B) Secondary antibody only. (C) After one hour incubation with labeled CPMV. (D) Vimentin surface staining of HeLa cells.
