# Supplementary material for: Endothelial Targeting of Cowpea Mosaic Virus (CPMV) via Surface Vimentin
Source: PLoS Pathog. 2009 May 1;5(5):e1000417. doi: 10.1371/journal.ppat.1000417 (PMC2670497; doi:10.1371/journal.ppat.1000417)
Supplement: Figure S6 — CPMV and CD31/PECAM (an endothelial marker) co-localize on the lumenal surface of mouse aorta. An adult C57Bl6J mouse was intravenously inoculated with Alexafluor 555-labeled CPMV. After one hour the mouse was anesthetized, aorta perfused with PBS and removed. From the freshly isolated aorta segments 10 µm cryosections were prepared and then cryosections were stained with CD31/PECAM antibodies. Blue = nuclei (DAPI), green = CD31/PECAM (A and C), red = CPMV (B and C), white = colocalization (C and D), * = lumen, and scale bar = 25 µm. (0.11 MB PDF) [file ppat.1000417.s006.pdf]

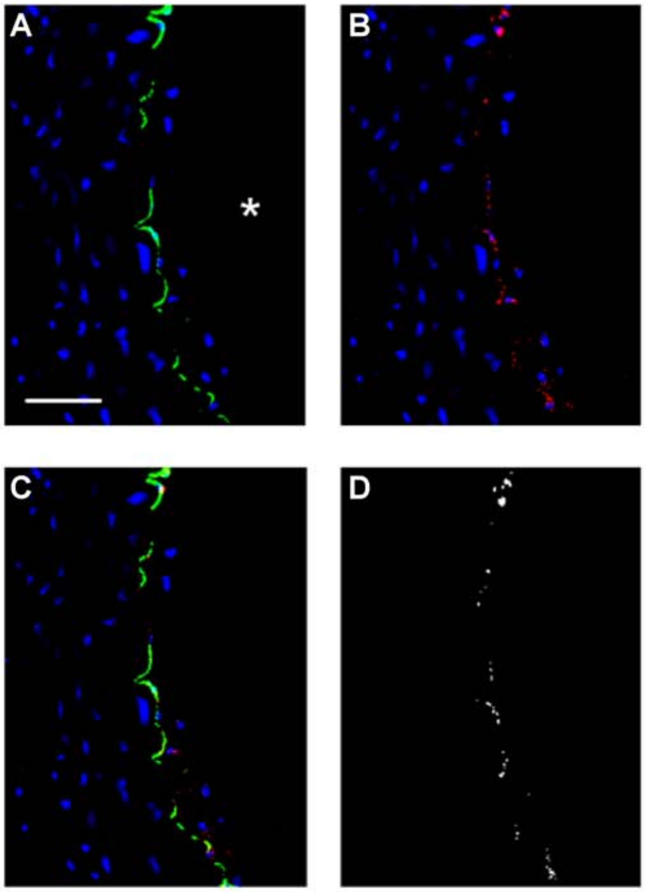

Figure S6: CPMV and CD31/PECAM (an endothelial marker) co-localize on the lumenal surface of mouse aorta. An adult C57Bl6J mouse was intravenously inoculated with Alexafluor 555-labeled CPMV. After one hour the mouse was anesthetized, aorta perfused with PBS and removed. From the freshly isolated aorta segments 10µm cryosections were prepared and then cryosections were stained with CD31/PECAM antibodies. Blue = nuclei (DAPI), green = CD31/PECAM (A and C), red = CPMV (B and C), white = colocalization (D), * = lumen, and scale bar = 25µm.
