# Supplementary material for: Endothelial Targeting of Cowpea Mosaic Virus (CPMV) via Surface Vimentin
Source: PLoS Pathog. 2009 May 1;5(5):e1000417. doi: 10.1371/journal.ppat.1000417 (PMC2670497; doi:10.1371/journal.ppat.1000417)
Supplement: Figure S7 — Additional examples of CPMV and surface vimentin co-localization on the lumenal surface of rat aorta evaluated by confocal microscopy. (A–E) Freshly isolated rat aorta was incubated with CPMV and vimentin antibodies ex vivo, and 10 µm cryosections made. Blue = DAPI, green = vimentin, red = CPMV, white = colocalization, * = vessel lumen, and bar = 100 µm (A) and 25 µm (B) respectively. (0.10 MB PDF) [file ppat.1000417.s007.pdf]

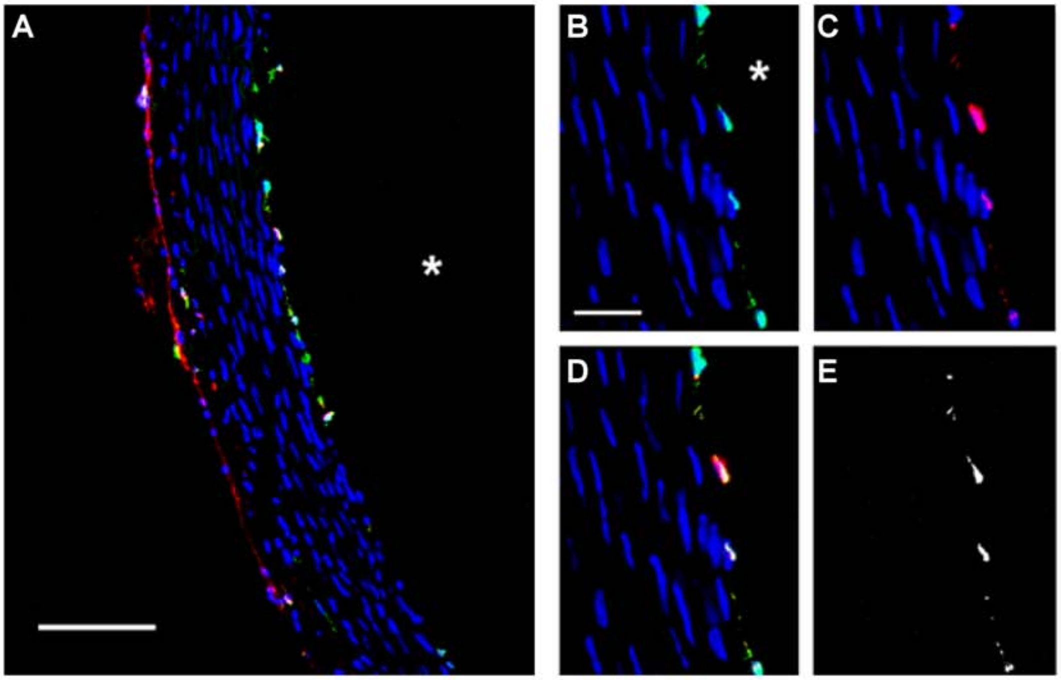

Figure S7: Additional examples of CPMV and surface vimentin co-localization on the lumenal surface of rat aorta evaluated by confocal microscopy. (A-E) Freshly isolated rat aorta was incubated with CPMV and vimentin antibodies ex vivo, and 10μm cryosections made. Blue = DAPI, green = vimentin, red = CPMV, white = colocalization, * = vessel lumen, and bar = 100μm (A) and 25μm (B) respectively.
