# Supplementary material for: Endothelial Targeting of Cowpea Mosaic Virus (CPMV) via Surface Vimentin
Source: PLoS Pathog. 2009 May 1;5(5):e1000417. doi: 10.1371/journal.ppat.1000417 (PMC2670497; doi:10.1371/journal.ppat.1000417)
Supplement: Figure S8 — Establishing specificity of CPMV and vimentin staining in rat aorta via fluorescence confocal microscopy. Rat aortic segments incubated ex vivo with labeled CPMV or vimentin-specific antibodies were cryosectioned and stained with secondary antibodies. (A) CPMV. (B) Monoclonal anti-vimentin antibody. (C) Monoclonal isotype control. Bar = 10 µm, * = lumen. (0.09 MB PDF) [file ppat.1000417.s008.pdf]

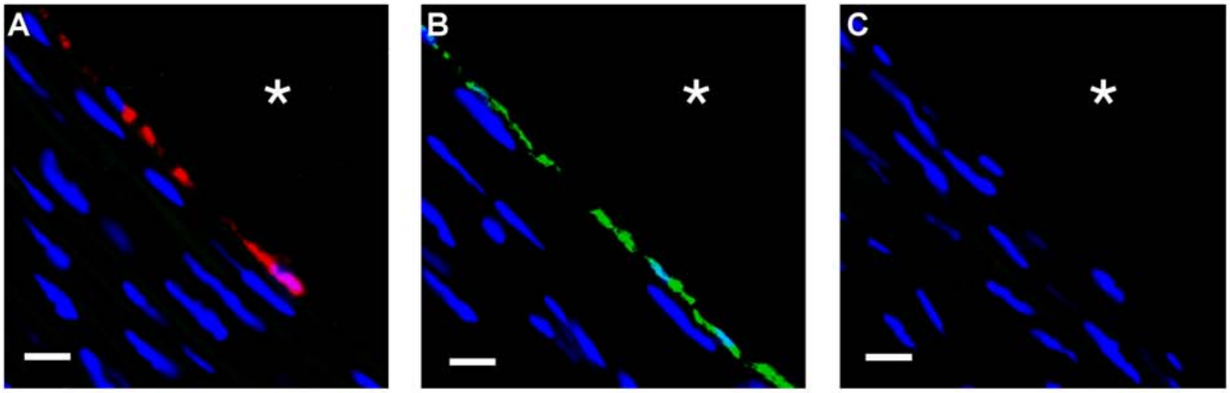

Figure S8: Establishing specificity of CPMV and vimentin staining in rat aorta via fluorescence confocal microscopy. Rat aortic segments incubated ex vivo with labeled CPMV or vimentin-specific antibodies were cryosectioned and stained with secondary antibodies. (A) CPMV. (B) Monoclonal anti-vimentin antibody. (C) Monoclonal isotype control. Bar = 10μm, *= lumen.
